# A Memoir of James Jackson, Jun. M.D.; with Extracts from His Letters to His Father, and Medical Cases Collected by Him

**Published:** 1836-01

**Authors:** 


Art. X.—*A Memoir of* James Jackson, Jun. m.d.; *with Extracts from his Letters to his Father, and Medical Cases collected by him.* By James Jackson, m.d., Professor of the Theory and Practice of Physic in Harvard University; and Physician of the Massachusetts General Hospital.—*Boston,* (*U. S.*) 1835. 8vo. pp. 444.

On this affecting volume,—for such it is,—we could, we think, compose a long article, not uninteresting and not uninstructive to the reader; but our limits warn us that we can but allude to its various and mournful, and yet pleasing contents. The story which it contains is sad, and soon told: that of an ardent and intelligent youth, the hope of his parents and of society, pursuing his studies in America and in Europe, so as to attract not the regards only, but the friendship of his teachers, and the warm esteem and admiration of many friends among his seniors, and those of his own age; passing from those studies to what was expected to be the scene of his future labours, full of energy, and animated by noble views, and cheered by happy prospects; the pride of his father, and an honour to the country of his birth; and, in a few short months, all those commencing labours interrupted by sickness, and all those hopes, and that just paternal pride, and every worldly aspiration and prospect closed by death:—adding one more to the many examples of human insecurity, which deprive reflecting persons of all solid hope except what points to a less frail and transient existence.

If any thing could add to the interest of a narrative of such events, it is that this record is made by the bereaved father, who has thus raised an imperishable monument to a son of whom any father must have felt proud. Every page tells of the recent life, the early death, the sorrow of those surviving. The dedication is to M. Louis and to Dr. Boott, "the two men to whom, among many, he (the deceased) felt most indebted whilst in Europe;" to M. Louis, whom he "regarded as a second father;" to Dr. Boott, "whose bright mind and pure and elevated virtues inspired the most ardent and sincere love in his young friend." It is indeed a sufficient eulogium of the young American, that he was honoured with friendship and kindness by men like these. We ourselves had the pleasure of only a very slight acquaintance with Dr. Jackson, jun., but this was sufficient to impress us with a high opinion of his merits, and we know that, in the judgment of some of the first physicians in London, he was considered as being a model to medical students.

The whole of the short life of the subject of the memoir, his thoughts, his hopes, his labours, are concentrated in this volume. Its few and simple incidents are related candidly, and without affectation; his merits are dwelt upon without exaggeration; and his own letters, the cases collected by him during his studies, and his own remarks upon them, justify every syllable that is said in his praise. Nor can we refrain from expressing our admiration of the firm and resigned spirit in which the task of collecting these remains has been performed by Dr. Jackson, sen. "In some points," he says, "the task has been grateful to me; sad though it may seem for a father." "I thank God," he adds, "that I have been able to maintain my cheerfulness, and to attend to the common occupations of life, since the deplorable loss which I suffered in his departure from this world. But every hour has he been in my mind. In every occupation, in almost every conversation, however little others could see the connexion, his image has been before me. It has been a beautiful image, and has not checked any pleasure, nor even any gaiety in which I thought that he could have joined." These are the accents of a manly and sacred grief, such as no father, we apprehend, can listen to unmoved.

In one comprehensive paragraph we learn the dates and chief events of the life of Dr. James Jackson, jun.

"He was born on the 15th January, 1810, was graduated at the University in Cambridge" (U. S.) "in 1828, and then engaged in the study of medicine. This he did under my direction, and as my pupil. He continued as such till the April of 1831, and during this time he attended the medical lectures of our university, and saw the practice of the Massachusetts General Hospital. In the spring, 1831, he went to Paris, where he arrived in May, and remained till July, 1833, except during a visit of six months to Great Britain and Ireland, in the spring and summer of 1832. He reached home at the end of the summer, 1833; and was graduated as doctor of medicine in our university, in February, 1834. He was now prepared to engage in practice, and took rooms for himself in Franklin-place. He was thus brought to the starting-place of active life, and under circumstances the most flattering and the most grateful, when he was arrested in his course. Exactly at this point he was arrested. His arrangements being made, he sent an advertisement to the public papers, which appeared on the 5th of March, and on that day he was taken sick, so as to lodge at my house instead of occupying the rooms which he had just announced as his residence. This sickness was his last, and he died on the 27th of the same month, being in his twenty-fifth year." (P. 4.)

He appears to have possessed great vivacity, great disinterestedness, a most warm and friendly disposition, and a singular aptitude for study. Before the second year of his medical studies was completed, he wrote a dissertation on Pneumonia, which so evinced his research as to gain him the Boylston medical prize. The extract given of a letter from his excellent father to him, when setting out for Europe, gives assurance that the heart of the son must have been ever touched and influenced by the example of a parent at once so affectionate and so judicious. "There is a risk of life," says this excellent man, "and it would indeed alter the aspect of my future days if I did not hope to have you by my side, and to leave you behind me in this world. But this is the smallest risk by far. Whether we pass a few short years together in this world, is comparatively of little consequence: whether we meet in a better world, is of immeasurable importance. This depends on ourselves,—on the strict regard to morality which we both maintain; a morality in Dr. Holyoke's sense, which includes piety,—a regard to our Maker, as well as to ourselves and fellow-men." Again, how simple, and yet how impressive, is the following expression: "In temptation, I think you will first think of home, and then cast your eyes higher,—to the home we all ultimately hope for, and to the Father who is better than any earthly parent." Well might his noble son say in reply, "My heart beats, and my eyes fill, and my hopes are brightened, as I advance in reading your kind letter of affection and advice." Nothing, indeed, can be more beautiful than those farewell letters between such a father and such a son. We wish that every English student would peruse and reflect upon them.

All the parent's fondest hopes were realized by his son's conduct and progress on his arrival in Paris, where his industry acquired new force from the examples of diligence by which he was surrounded; and his zeal for knowledge, together with his excellent principles, secured him from all the common temptations met with in great cities. Without any special introduction to them, he became the favourite pupil and personal friend of M. Louis and M. Andral; a circumstance which speaks most strongly for his application and intelligence. M. Louis became his correspondent after his return to America, and appears to have entertained the highest regard for him. It is indeed very delightful to us, who have so often to refer to the professional labours of M. Louis, to find in this memoir convincing proofs that his great talents, his astonishing industry, and his perseverance in the investigation of medical truths, is combined with every quality that can endear him to those who are honoured with his friendship. The extracts given from his letters, in which he makes some suggestions as to the future course to be pursued by Dr. Jackson, are, as may be supposed, extremely interesting. It is evident that he thought most highly of the pupil's powers of observation; and he strongly urged the devotion of four or five years to their exclusive employment. When we read the earnest and friendly suggestions of this accomplished Frenchman to the father of a young American student, it seems to us as if the natural boundaries of countries were no more felt in the pursuit of science, but that a bond of union and peace was forming, as we trust it is, which meaner considerations could never break or destroy.

In the account given of the Parisian life of young Jackson, we can see an explanation of the attachment shewn to him by the most eminent men of the medical school of Paris. During a residence of eighteen months, he devoted not less than five hours, and sometimes seven hours, a day to the hospitals; a great portion of which time was employed in the interrogation and examination of patients, or in investigating appearances after death. The remainder of the day was filled up with attention to practical anatomy, to obstetrics, and to the society of those whose professional knowledge was likely to increase his own. His letters bear continual testimony to his unremitting labours, and to his anxious desire to learn and to improve. The valuable hints derived in the morning's conversation with Louis or Andral were often subjected, before the day closed, to trial at the bedside of the hospital patients. Of some of the eloquent lectures of Andral he sent his father faithful abstracts, evidently made before any part of the impression had faded. It is impossible to read his almost daily records without being carried into the very midst of the Parisian school, and beholding all the energies by which it is characterized, as it were displayed. Some of our London students might see, in this kind of diary, a somewhat striking contrast to that apathy which is contented with the mere knowledge that will meet the common emergencies of country practice, and which no desire to extend he boundaries of medical science ever disturbs.

It was our intention to give several extracts from Dr. Jackson, jun.'s Letters, which will probably not be seen by many students in the original memoir. We can, however, only afford space for one or two. The following conveys some idea of the manner in which the teachers of Paris interest their followers, and is at the same time characteristic of the industrious writer:

"*Paris; November* 28, 1831.

"I am still following at la Pitié. I have made two or three efforts to follow Chomel at Hôtel Dieu; but it is impossible to do so with advantage. One may hear the clinique, to be sure, and a very good one too; but one cannot see the patients. This, especially in my present situation, is the most important by far. My great object is to accustom my ear to stethoscopic sounds: in order to do this I must see the patients. The visit at Hôtel Dieu is commenced an hour and a half before clear daylight, by candle-light, indeed;—there are from two to three hundred pupils in the wards at the same time, and one is fortunate if he sees four patients, and examines one, in the course of the visit. Whereas, the visit is made at la Pitié by daylight; there are not more than fifteen students, and I call it a black day in which I have not examined as many as six patients at least, who present stethoscopic phenomena; ordinarily I examine as many as ten. Besides, as I have told you before, Louis gives a little clinique at each bed. You see that I do right in giving the preference to la Pitié. I think I am becoming daily more able to distinguish the signs which indicate commencing phthisis. They are not one, but many.

"I find that both Louis and Andral depend more upon the respiration, its force, or its modification, the râles, craquement, gargouillement, and the sound when the patient coughs, than upon the voice. The craquement, or, when more marked, gargouillement, is, with them, a very important sign. When slight, it indicates the commencing ramollissement. Frequently it is not heard during the common respiration, but only when the patient takes a full inspiration, or coughs. This, if attended with obscurity, or feebleness in the respiratory murmur, is very decisive. Andral says, in as many words, in an article on auscultation, which he has lately written in the new Dictionary of Medicine, that he depends much more upon the respiration, the râles, and the sound of the cough, as affording evidence of tubercles, than upon the voice. In this same article he makes some very important and very interesting distinctions, especially on the subject of bronchial respiration. I think he has facilitated for me the study of auscultation with children, particularly by his description of one variety of bronchial respiration. I fancy that one or two of those mistakes in diagnosis, of which I lately wrote you, were owing to a want of attention on my part to this distinction. If so, I may hope to profit by them. Since reading the article, I have not yet had an opportunity to settle this important question." (P. 88.)

The account given of the first appearance of the cholera in Paris, in March 1832, of its rapid spreading through the city, of the crowded state of the wards, and of the dismay communicated to the minds even of experienced physicians by the presence among them of this awful disorder, is extremely graphic; being, indeed, a simple and natural detail of the events as they occurred, and of the immediate impression made, and far more striking than the most highly-wrought description. His desire to study the disease, and his efforts to relieve the anxiety of his father, produced a struggle in his mind at this time which is very forcibly portrayed in several of his letters. He determined, rightly we think, not to leave the post of danger; and pursued his clinical observations with new interest and fresh industry. "For the disease,"—he says, "one word;—it is death. Truly, at Hôtel Dieu, where I have seen fifty and more in a ward, it is almost like walking through an autopsy room—in many nothing but the act of respiration shows that life still exists; it is truly awful." Eight days afterward, (April 8,) he says, "I am with Andral. During five days we have had eleven very exact autopsies," &c. &c. "I work harder than ever in my life before." "I shall send you one hundred detailed observations, and forty or fifty of the most thorough and accurate autopsies that you ever read." In another letter, written on his way to London, some weeks afterward, he says, still speaking of the disease he had lately lived amidst: "you have no conception of the mortality; and allow me, your son and pupil, to say to my father and master, you have no conception of the disease, and will not have till you have seen it. The Frenchmen even, who look upon death and dying with as much sang froid as any people, were thrown off their balance. Never shall I forget Louis's altered face and aspect for the first week, emaciated, wan, wretched, like one who had received a blow from which he had not recovered. There are few men living so familiar with death or the dead."

We refrain, with reluctance, from following our young and intelligent traveller to England, where it is gratifying to perceive that the public institutions and collections, especially the Hunterian, afforded him great interest. Of the museum at Guy's hospital, and of Dr. Hodgkin, he speaks in deserved terms of praise. In a tour through some of the midland and northern provinces, and part of Scotland and Ireland, he alludes every where to the kindness and hospitality which was shewn to him. Our American friends will see, we hope, in this part of Dr. Jackson's travels, a proof of the liberal manner in which they are sure to be received by the most esteemed members of our profession in England, Ireland, and Scotland. The love of science is too pure and elevating to permit those imbued with it to be contaminated by the narrower and baser feelings, so mischievously encouraged by too many writers who have visited America, and who have composed books apparently without entertaining any views worthy of the people of an enlightened country.

We must also pass over Dr. Jackson's second visit to Paris; and we are unable to notice the numerous cases appended to the Memoir and Letters. Indeed, we should be glad to see the letters and the memoir, somewhat abbreviated, and more carefully arranged, published in a small volume, without the cases. Students have too few of such books, and think too little of the noble examples which have adorned the profession of medicine.

Little more remains to be said. Dr. Jackson returned to his father and to his home, and prepared to fulfil as a practitioner and a citizen all the hopes to which his virtues as a student had given rise. Whilst studying the autumnal fever, as shewn in the Massachusetts hospital, and tracing its analogies with the fever of Paris, so well described by his friend and preceptor Louis, he was himself seized with the disease, and, partly perhaps in consequence of the existence of previous intestinal irritation brought on during his great exertions in Paris, suffered from it very severely. Diarrhœa remained during his convalescence; dysentery supervened; and, seven months after his return to New York from Europe, he ceased to exist.

We wish that our fervent testimony to the merits of this excellent young man could convey to his father some trifling consolation in such an irreparable calamity: but we think he does not need it. His letters, and the composition of his memoir, shew that his mind is carefully regulated, and that his hopes are not merely worldly. To such a mind, even in the deepest distress occasioned by such a trial, the reflection must soon have presented itself that the merits of his excellent son, his duties, and his virtues, would not be measured by the mere limits of his earthly life. Young as that son was, he left an example not likely to be lost upon the aspiring young men of his own great country; and we can truly add, that it is one well worthy of being followed by every European student whose wish it is to obtain honourable distinction, and pure and permanent fame.

